# Anti-Factor H Antibodies in Egyptian Children with Hemolytic Uremic Syndrome

**DOI:** 10.1155/2021/6904858

**Published:** 2021-11-18

**Authors:** Shereen Shawky, Hesham Safouh, Mona Gamal, Mohammed M. Abbas, Azza Aboul-Enein, Toshihiro Sawai, Yosra Fahmy, Heba Selim

**Affiliations:** ^1^Department of Clinical and Chemical Pathology, Faculty of Medicine, Cairo University, Cairo, Egypt; ^2^Department of Pediatrics, Faculty of Medicine, Cairo University, Cairo, Egypt; ^3^Department of Clinical and Chemical Pathology, Faculty of Medicine, Fayoum University, Fayoum, Egypt; ^4^Department of Pediatrics, Shiga University of Medical Science, Seta-Tsukinowa, Otsu, Japan

## Abstract

**Background:**

Atypical hemolytic uremic syndrome (aHUS) is an important cause of acute kidney injury in children. It is primarily caused by dysregulation of the complement alternative pathway due to genetic mutations, mainly in complement factor H genes, or due to anti-factor H autoantibodies (anti-FH), leading to uncontrolled overactivation of the complement system. Early diagnosis and treatment of autoimmune HUS (AI-HUS) is essential and leads to a favorable outcome.

**Methods:**

Fifty pediatric HUS patients and 50 age- and sex-matched controls were included in the study. Patients were subjected to full history taking, clinical examination, and laboratory testing. All candidates were subjected to an assessment of anti-FH in serum by a homemade enzyme-linked immunosorbent assay technique.

**Results:**

A high frequency of serum anti-FH was detected in our aHUS patients. The disease onset of AI-HUS was mainly observed in March and April, with significantly higher rates in school-aged males. All patients who started immunosuppressives early together with plasmapheresis upon detection of their anti-FH had complete renal function recovery.

**Conclusion:**

The high frequency of AI-HUS revealed in Egyptian HUS children in our study highlights the importance of implementing anti-FH testing in Egypt to provide early recognition for immediate proper management, including early immunosuppressive therapy, and hence improving patient outcomes.

## 1. Introduction

Thrombotic microangiopathy (TMA) defines a group of diseases characterized by microangiopathic hemolytic anemia (MAHA), thrombocytopenia, and organ injury [1]. TMAs resulting in acute kidney injury are referred to as hemolytic uremic syndrome (HUS). Most HUS cases are caused by infection with Shiga toxin-producing *Escherichia coli* (STEC), while nearly 10%, referred to as atypical HUS (aHUS), are associated with uncontrolled activation of the alternative complement pathway (ACP) [2].

aHUS may be due to genetic mutations affecting the genes encoding complement regulatory proteins, more frequently, complement factor H (CFH). In addition, acquired functional CFH deficiency due to anti-factor H autoantibodies (anti-FH) has been observed and termed autoimmune HUS (AI-HUS) [3]. Anti-FH inhibits the regulatory function of CFH at cell surfaces by binding mainly to epitopes within the C-terminus, thus preventing it from interacting with C3b, C3d, and heparin and thereby diminishing the protection of host cells against complement attack. Anti-FH might also bind to the CFH N-terminus and middle part, thereby markedly weakening its interactions and interfering with factor I cofactor activity, possibly leading to the neutralization of all CFH functions and causing more severe disease forms [4].

aHUS differs from STEC-HUS in having worse outcomes and poorer prognosis, as well as higher recurrence rates following kidney transplantation [5].

In the current study, we aimed at studying the frequency of anti-FH as a contributing factor for the development of aHUS in Egyptian children and to explore its relation to disease severity and outcome.

## 2. Materials and Methods

### 2.1. Patients

Fifty HUS patients were recruited from the Pediatric Department of Cairo University Children Hospital between March 2018 and July 2019. Patients fulfilled the clinical and laboratory criteria consistent with HUS including the presence of MAHA and thrombocytopenia (hematocrit <30%, hemoglobin level (Hb) < 10 g/dl, serum (s) lactate dehydrogenase (LDH) > 500 U/l, presence of peripheral blood (PB) schistocytes, and platelets <150,000/mm^3^ associated with acute kidney injury (s.creatinine >0.8 mg/dl for children aged 5–10 years and >0.5 mg/dl under the age of 5) [6–8]. Patients were tested for disintegrin and metalloproteinase with a thrombospondin type 1 motif, member 13 (ADAMTS13) levels by ELISA (Abcam, Cambridge, UK) to exclude the diagnosis of thrombotic thrombocytopenic purpura (TTP). HUS patients who have already started treatment with fresh frozen plasma (FFP) or plasma exchange (PEX) were excluded. Fifty age- and sex-matched healthy children were included as controls. Our study was approved by the ethical committee of the Faculty of Medicine, Cairo University, and in accordance with the Helsinki Declaration. Informed consent was obtained from the parents of all subjects included in the study.

## 3. Methodology

Clinical and demographic data were collected for all patients. Laboratory investigations included Shiga toxin assay (ELISA) (MyBioSource, San Diego, USA), complete blood counts, renal function tests, LDH, and haptoglobin. The complement system was evaluated by measuring serum C3, C4, and CFH by ELISA (Hycult Biotech, the Netherlands). Management and outcome data were recorded during follow-up at least for 3 months.

### 3.1. Measurement of Serum Anti-FH

Anti-FH was measured in all subjects by a homemade indirect ELISA technique, as described previously by Sénant and Dragon-Durey and by Mousavi et al. [9, 10]. Both the purified CFH material and the anti-FH standard were provided by Dr. Toshihiro Sawai (Associate Professor, Department of Pediatrics, Shiga University of Medical Science, Japan).

In brief, ELISA plate microwells were coated with 50 *μ*L of diluted purified CFH at a concentration of10 *μ*g/ml in each well using 0.1 M NaHCO3 at PH 9 for dilution. The sealed microplate was left overnight at +4°C and then washed twice using TBS wash buffer (Tris-buffered saline/0.2% Tween 20), followed by plate blocking using 200 uL TBS/BSA 1% blocking/working solution (Tris-buffered saline/0.2% Tween 20 + 1% bovine serum albumin) and incubating again for 1 hour at 37°C. 50 *μ*L/well of 1/50 diluted patients' samples in the working solution were then dispensed. Simultaneously, a blank and 50 *μ*l/well of twofold serial dilutions of the standard sample from 1/800 to 1/51200 were added to the first 7 wells (corresponding to 1400, 700, 350, 175, 88, 44, and 22 AU/ml, respectively). The microplate was kept for 1 hour at room temperature and then washed. 100 *μ*L of 1/2500 diluted anti-IgG conjugate labeled with horseradish peroxidase were dispensed and incubated for 30 minutes at room temperature, and 100 *μ*L substrate solution (OPD) was added for 3 to 5 minutes at room temperature. Results were read on a microplate reader at 492 nm (a 690 nm filter was used as a reference) after adding 50 *μ*l/well of 1M H2SO4 stop solution.

### 3.2. Statistical Analysis

Data were collected, coded, and entered into Microsoft Access. All analyses were performed using the Statistical Package for the Social Sciences software 17 (SPSS Inc., Chicago, USA). For quantitative parametric data, the independent Student's *t*-test was used to compare two independent groups, while one-way ANOVA test was used in comparing more than two groups. For qualitative data, chi-square test was used. Association studies were performed using Spearman correlation analysis.

## 4. Results

Of the 50 HUS patients, 28 (56%) were males and 22 (44%) were females, with a mean age of 4.1 ± 3.6 years (range, 6 months to 12 years). Nine patients (18%) came from consanguineous marriages. The 50 controls were of matched age (6.2 ± 2.8 years) and sex (30 males and 20 females).

According to the clinical and laboratory findings, patients were grouped into 3 groups:

- 22 typical HUS patients (44%) due to STEC infection evidenced by a positive Shiga toxin assay
- 12 aHUS patients (24%) were positive for serum anti-FH
- 16 aHUS patients (32%) were negative for anti-FH and showed no evidence of STEC infection

### 4.1. Demographic Data and Clinical Findings

The demographic and clinical data are presented in Table 1. The main presenting symptoms were as follows: bloody diarrhea in 22 patients (44%) and watery diarrhea in 15 patients (30%), vomiting in 42 patients (84%), and fever in 33 patients (66%), and 8 patients (16%) had chest infections. The clinical characteristics were oliguria/anuria in all patients, generalized edema in 35 patients (70%), jaundice in 10 patients (20%), and CNS affection (disturbed consciousness and/or convulsions) in 50% of the patients.

In patients with anti-FH, the percentage of males was significantly higher compared to the anti-FH-negative aHUS group (*p*=0.02). The age of patients was significantly higher in anti-FH-positive patients (7.7 ± 3 years) compared to typical HUS (1.95 ± 1.6 years) and anti-FH-negative aHUS groups (4.3 ± 4 years) (*p* < 0.001 and 0.02, respectively). Consanguinity rates were similar among different patient groups (*p* > 0.05).

Disease onset in the anti-FH-positive patients showed a peak incidence in March and April (58.3%), unlike the other patient groups. There was no significant difference regarding the preceding illness and clinical features between different patient groups, except for a lower incidence of diarrhea and fever in the anti-FH-positive patients compared to the typical HUS group (*p*=0.002 and 0.04, respectively).

### 4.2. Laboratory Findings

All patients showed laboratory manifestations of MAHA including low Hb, PB, schistocytes >2%, increased reticulocytes, reduced haptoglobin, increased LDH, and thrombocytopenia, in addition to impaired renal function tests, and all of them had normal ADAMTS13 levels. Our patients with anti-FH showed significantly lower platelet counts and higher LDH than the antibody-negative aHUS patients (*p*=0.006 and 0.02, respectively). Although a negative correlation was observed between C3 levels and anti-FH titers in our patients, it was statistically insignificant. The percentage of patients with low serum C3 and C4 was comparable between aHUS patients with and without anti-FH (*p* > 0.05) (Table 2).

### 4.3. Anti-FH Abs Levels

The positive threshold for the anti-FH titer in our study was set up as the mean antibody level +2 standard deviation (SD) measured in sera of 50 normal controls and was specified accordingly as 250 AU/ml. The specificity of all positive sera (>250 AU/ml) was confirmed by assay replication. 12 of our 28 aHUS patients (42.9%) were diagnosed as having anti-FH-associated aHUS with a mean anti-FH titer of 3440 ± 2720 AU/mL (range: 400–8350 AU/mL). Regarding serum CFH in our patients, reduced levels were detected in only 2 (16.7%) of the anti-FH-positive patients, which was insignificantly different from other HUS patients (Table 3).

### 4.4. Treatment Regimens and Different Outcomes

Therapy regimens and outcome data of the first flare for at least 3 months of follow-up are presented in Table 4. At the disease onset, the treatments of the 12 patients with anti-FH were as follows: 10 patients (83.4%) needed renal dialysis, 4 patients (33.3%) received FFP only, and PEX ± FFP transfusions were performed in 3 patients (25%). Following the release of our anti-FH assay results, 5 other patients (41.7%) received both PEX and induction immunosuppression (IS), including steroids alone (*n* = 3), steroids combined with cyclophosphamide (*n* = 1), or mycophenolate (*n* = 1). IS was started early during the first two weeks in 4 children, while the fifth patient started steroids late, about a month of starting PEX treatment.

The outcome was described as favorable when complete remission (CR) was achieved or adverse when renal sequelae such as chronic renal failure (CRF) or extrarenal sequelae such as CNS and intestinal complications or even patient death occurred. CR was defined by renal function recovery (glomerular filtration rate >80 mL/min/1.73 m^2^, without proteinuria) plus normalization of hematologic parameters (Hb > 10 g/dl, platelets  > 150 × 10^9^/L, and LDH < 450 U/L). The clinical outcomes of the 12 patients with anti-FH were as follows: 6 patients (50%) achieved CR, 4 patients (33.3%) had CRF, and 2 patients (16.7%) developed other organ complications; one of whom unfortunately (8.3%) died. No significant difference was observed in the mean anti-FH titers between different disease outcomes within the anti-FH-positive group (*p* > 0.05).

Analysis of the different clinical outcomes in patients with anti-FH in relation to different treatment regimens showed that patients who started IS early together with PEX (*n* = 4) had better renal recovery with no other organ complications compared to those who started IS late as well as the other patients who received only FFP/PEX. Thus, 4 out of 5 children who received IS achieved CR, representing 80% compared to only 2 patients achieving CR out of 7 anti-FH-associated patients (28.6%) who received only FFP/PEX. Combined therapy with PEX and IS was strongly associated with favorable outcome (OR: 0.23, 75% CI: 0.08–0.84; *P*=0.026). One of the 3 anti-FH-positive patients who received PEX ± FFP achieved recovery; one had CRF while the third patient developed cardiac muscle insufficiency. Only one of the 4 patients who received only FFP recovered, 2 patients developed CRF, and one died of cardiac failure.

## 5. Discussion

Autoimmune HUS is a severe, multisystem disease with serious presentation. Most AI-HUS patients show marked anemia, thrombocytopenia, and severe renal affection with poor outcomes and increased rates of recurrence, morbidity, and mortality [5].

The identification of aHUS patients with anti-FH is important to target these patients with proper therapy, predict organ involvement, monitor relapses, guide appropriate follow-up, and adjust treatment accordingly. Management of AI-HUS consists mainly of PEX, eculizumab therapy to control complement system activation, and immunosuppressives such as prednisolone and other agents to suppress the autoantibody load [2]. Considering that eculizumab is an expensive drug, it is unlikely to be available soon for routine use for these cases in developing countries [11]. Fortunately, the AI-HUS is reported to have excellent results with a combination of PEX and IS drugs [6]. Thus, prompt PEX with early administered IS drugs remains the chief management of choice for AI-HUS, especially in developing countries. However, early diagnosis and treatment of this specific entity of aHUS represents a cornerstone in the efficient management aiming at improving disease outcomes in these cases [12].

Herein, we have developed and optimized a homemade ELISA kit for the detection of anti-FHs antibodies as it is highly required due to the shortage of commercial testing kits. Using this test assay, we evaluated the occurrence of anti-FH in Egyptian children with aHUS. Up to our knowledge, this is the first study to test for the presence of anti-FHs in HUS patients in Egypt.

In the present study, an increased frequency of serum anti-FH-positive cases was detected in our aHUS cohort (42.9% of aHUS patients). This finding highlights the increased prevalence of AI-HUS in Egyptian children. The high frequency observed is nearly consistent with other figures reported from India, estimating a frequency of around 55% [13, 14]. On the other hand, published data from studies conducted in different European countries reported much lower frequencies of circulating anti-FHs (5–25%) [8, 15, 16].

The reason for the increased frequency of anti-FH-associated HUS observed in our study is unclear.

Several theories have been previously postulated for explanation. In published studies conducted in India, an increased frequency of CFH-related proteins 1–3 (CFHR1–R3) deletions has been linked to the development of anti-FHs. They assumed that due to the structural similarity between the CFH C-terminal domain and the CFHR1 C-terminus, most anti-FHs cross-react with short consensus repeats (SCRs) 4-5 of CFHR1, suggesting that the absence of CFHR1 plays a role in the loss of immune tolerance to CFH [17, 18]. This explanation, however, was not satisfactory to explain the dramatically high prevalence of anti-FH-associated aHUS reported in Indian children and is attenuated by the fact that the population frequency of CFHR1 deletions is similar in India compared to other populations with lower incidence of anti-FHs [14, 16, 18, 19]. Moreover, anti-FH-associated aHUS is an ultrarare disorder with an estimated prevalence of about 1/1,000,000 people in the USA and Europe [18, 20], which does not fit with the approximate 5% prevalence of CFHR1 deficiency in European Caucasian populations [21]. The population frequency of CFHR deletions in Egypt was not determined before and still needs to be investigated in future studies. Other genetic factors, such as at-risk HLA haplotypes, might also be implicated in the increased frequency of AI-HUS [22].

In the present study, a significantly higher age of patients at disease onset was detected in patients with anti-FH compared to patients without. This difference supports the hypothesis that diverse risk factors predispose to the two forms of the disease. Our results agree with other published data, confirming that AI-HUS affects older children, especially school-aged children and adolescents [15, 23].

Our results show that many patients with anti-FHs had prodromal infectious illnesses, most commonly gastrointestinal and upper respiratory infections. In addition, a seasonal prevalence in March and April was noted. These findings, together with previously published data showing a high prevalence of prodromal infections with common pathogens in patients with anti-FH [24], are in favor of a “two-hit” model according to which the autoimmunity toward CFH could evolve due to infections in genetically predisposed subjects [18]. In line with this, Puraswani et al. stated that the high prevalence of the disease in school-going children together with a predilection for cold weather and associated prodromal symptoms reflects a possible infectious trigger [14]. Microbes bind CFH via an anti-FH epitope cluster within SCR20, inducing conformational alteration, which generates a neoepitope similar to FHR1 and finally predisposes to the development of anti-FH in individuals with FHR1 deficiency [19].

In this study, patients with anti-FHs had significantly lower platelet counts and higher serum LDH levels compared with those without, which might reflect a correlation of anti-FHs with disease severity. In conformity, it was reported that anti-FH titers were related to disease severity as evidenced by a relationship with platelet counts, hemoglobin levels, LDH levels, and dialysis requirements [14]. It is noteworthy, however, that hemoglobin and S.creatinine levels showed no significant difference in our aHUS patients with and without anti-FH. This warrants further studies on a greater number of AI-HUS children to give a clearer picture about the significance of anti-FH as markers of disease severity in aHUS.

Serum CFH levels can be affected by the presence of circulating immune complexes of CFH. These complexes are expected to reduce the amount of free functional circulating CFH. Interestingly, reduced CFH levels were detected in only 2 of our 12 (16.7%) anti-FH-associated aHUS cases. This is close to data previously reported by Brocklebank et al., who found reduced CFH in 11.8% of their patients [15] while others showed normal levels [8, 23]. These data might be explained by the nature of the antibodies used in the CFH ELISA assay that possibly detects total CFH (including both free and immune-complexed forms).

About 25% of our anti-FH-positive patients had reduced C3 levels reflecting activation of the ACP. Similar figures (24–27%) were demonstrated in some studies [15, 16], while others stated much higher percentages [8, 25].

The evidence-based guidelines for the diagnosis and management of TMA recommended plasma therapy as the first-line management [26]. Thus, HUS patients admitted to the children's hospital during the acute phase were treated with the usual supportive management, consisting primarily of plasma transfusions and exchanges, diuretics, anticonvulsants, and renal dialysis when needed. Importantly, based on our results, 5 aHUS patients enrolled in the present study and with confirmed anti-FH (the results of which were released in the acute phase of the disease) started IS introduced for the first time in the treatment protocols of our aHUS patients in addition to prompt PEX sessions. Immunosuppressants were added according to experts' advice that AI-HUS patients should receive early prolonged PEX together with immunosuppressants to reduce antibody titers and supply complement factors [27, 28]. Our results support the previous recommendation reflected as better disease outcome at 3 months or more, in 4 out of these 5 children who received early IS combined with prompt PEX, compared to only 2 achieving CR out of 7 patients who received FFP/PEX only. Our results agree with others reporting a dramatic improvement in the prognosis of AI-HUS patients treated with combined IS and PEX therapy [2, 3, 28]. In line with this, Song et al. emphasized the benefits of plasma therapy combined with IS in anti-FH-associated HUS, especially in developing countries, but suggested that additional prospective evaluations are needed [28].

No significant correlation was revealed between anti-FH titers and prognosis in AI-HUS, which is in agreement with others [13]. This is, however, inconsistent with some published data suggesting higher antibody levels being associated with adverse outcomes [29]. Recently, Puraswani et al. stated that antibody titers exceeding 8000 AU/ml were considered a risk factor for adverse outcomes [14]. All our cases had antibody levels of less than 8000 AU/ml except for one patient. However, it is interesting to mention that this patient (with an anti-FH titer of 8350 AU/ml) had his anti-FH detected, received steroids, and started PEX very early upon admission and had CR without any other sequelae.

Among the study limitations in this work are the small sample size, the short follow-up periods, and lack of serial measurements of anti-FH over the study period. In addition, as the main pathogenesis of aHUS is supposed to be unrestricted overactivation of the alternative pathway of complement, the study of genetic mutations in different members of the complement system in Egyptian children is highly recommended. Including a larger number of patients in a multicenter study and for longer periods of follow-up will help obtain detailed information about the occurrence of AI-HUS in Egypt and so better guidelines for management and consequent follow-up in these patients can be generated.

In conclusion, this work showed that Egypt potentially has one of the highest reported frequencies of AI-HUS. Hence, we highlight the importance of the rapid detection of anti-FH and make the assay readily available for children with aHUS, especially older ones. The proper diagnosis of AI-HUS and rapid initiation of appropriate treatment will give the patients the chance of escaping renal injury, relapses, and attaining better outcomes.

## Figures and Tables

**Table 1:** Demographic features and clinical findings in HUS patients.

Parameter	Typical HUS (*n* = 22)	Anti-FH-positive aHUS (*n* = 12)	Anti-FH-negative aHUS (*n* = 16)
Sex/males	12 (54.5%)	10 (83.3%)	6 (37.5%)
*p* value	*p*=0.9		
		*p*=**0.02**	

Age (years)	1.95 ± 1.6	7.7 ± 3	4.3 ± 4
	*p* ≤ **0.001**		
		*p*=**0.02**	

Consanguinity	2 (9.1%)	4 (33.3%)	3 (18.7%)
	*p*=0.9		
		*p*=0.7	

*Prodromes*			
Diarrhea	22 (100%)	5 (41.6%)	10 (62.6%)
	*p*=**0.002**		
		*p*=0.1	

Fever	18 (81.8%)	6 (50%)	9 (56.3%)
	*p*=**0.04**		
		*p*=0.5	

Vomiting	20 (90.9%)	10 (83.3%)	12 (75%)
	*p*=0.7		
		*p*=0.6	

Respiratory infection	2 (9.1%)	2 (16.7%)	4 (25%)
	*p*=0.1		
		*p*=0.7	

*Clinical symptoms*			
Oligo/anuria	22 (100%)	12 (100%)	16 (100%)
	*p*=0.9		
		*p*=0.2	

Hypertension	3 (13.6%)	2 (16.7%)	4 (25%)
	*p*=0.5		
		*p*=0.6	

Generalized edema	15 (68.2%)	7 (58.3%)	13 (81.2%)
	*p*=0.3		
		*p*=0.7	

Jaundice	3 (13.6%)	4 (33.3%)	3 (18.8%)
	*p*=0.7		
		*p*=0.1	

CNS affection	11 (50%)	5 (41.7%)	9 (56.3%)
	*p*=0.7		
		*p*=0.6	

Data are presented as the number of patients with the percentage in parentheses. Bold values are significant at *p* < 0.05. Analysis was done using chi-square test.

**Table 2:** Laboratory data of HUS patients.

Parameter	Typical aHUS (*n* = 22)	Anti-FH-positive aHUS (*n* = 12)	Anti-FH-negative aHUS (*n* = 16)
Hb (g/dL)	6.6 ± 1.5	7 ± 0.7	7.1 ± 1.2
*p* value	*p*=0.8		
		*p*=0.9	

WBCs (10 ^3/cmm)	14.3 ± 5.3	10.7 ± 3.4	10.5 ± 4.9
*p* value	*p*=0.2		
		*p*=0.9	

PLT (10̂ ^3/cmm)	98.7 ± 54.4	79.1 ± 34	101.6 ± 46.2
*p* value	*p*=**0.3**		
		*p*=**0.006**	

LDH (U/L)	2031.8 ± 1080.5	2020.2 ± 1263.7	1305.4 ± 1038.1
*p* value	*p*=0.8		
		*p*=**0.02**	

Bilirubin (mg/dL)	0.89 ± 0.069	1.3 ± 1	0.93 ± 1.3
*p* value	*p*=0.8		
		*p*=0.09	

Reticulocytes (%)	4.8 ± 2.1	5.8 ± 1.9	4.2 ± 2.2
*p* value	*p*=0.4		
		*p*=0.3	

Creatinine (mg/dL)	6.8 ± 4.7	6.2 ± 4.6	5.9 ± 3.5
*p* value	*p*=0.09		
		*p*=0.8	

BUN (mg/dL)	121.1 ± 80.8	126.6 ± 58.6	111.1 ± 60.5
*p* value	*p*=0.09		
		*p*=0.5	

Albumin (g/dL)	3.1 ± 0.48	3.2 ± 0.6	2.9 ± 0.85
*p* value	*p*=0.2		
		*p*=0.08	

Low C3 (normal 80–160 mg/dL)	—	3 (25%)	2 (12.5%)
		*p*=0.6	

Low C4 (normal 20–40 mg/dL)	—	3 (25%)	5 (31.3%)
		*p*=0.9	

Low CFH (normal 70–130 ug/mL)	—	2 (17%)	4 (25%)
		*p*=0.7	

Hb: hemoglobin; WBC: white blood cells; PLT: platelets; LDH; lactate dehydrogenase; BUN: blood urea nitrogen; CFH: complement factor H. Data are presented as the mean ± standard deviation or the number of patients with the percentage in brackets. Bold values are significant at *p* < 0.05. Analysis was done using *t*-test and chi-square test.

**Table 3:** Serum levels and correlations of anti-FH.

A: serum anti-FH levels (AU/ml) in patients and controls						
Groups	Mean ± SD (range) in AU	Controls (*n* = 50)		78.3 ± 86.6 (10–430)		
Anti-FH-positive HUS (*n* = 12)		3440 ± 2720.5 (400–8350)				
Anti-FH-negative HUS (*n* = 38)		71 ± 48.4 (10–190)				

B: correlations of anti-FH in the anti-FH-positive patients						
Parameter				Anti-FH titer		
				R		*p* value
C3	−0.49	0.08	C4	−0.31	0.3	
Hb level				−0.45		0.1
LDH level				−0.46		0.1
Platelet count				−0.36		0.2
Creatinine levels				−0.33		0.3
Factor H levels				0.08		0.7

Analysis was done using Spearman's correlation test.

**Table 4:** Treatment regimens and different outcomes among HUS patients.

Parameter	Typical HUS (*n* = 22)	Anti-FH-positive aHUS (*n* = 12)	Anti-FH-negative aHUS (*n* = 16)
A: treatment regimens			
Renal dialysis	15 (68.2%)	10 (83.4%)	12 (75.1%)
PEX ± FFP	0	3 (25%)	6 (37.5%)
FFP transfusion only	1 (4.5%)	4 (33.3%)	10 (62.5%)
Combined PEX + IS other drugs	0	5 (41.7%)	0
Diuretics	21 (95.5%)	11 (91.7%)	15 (93.8%)
Anticonvulsants	1 (4.5%)	0	1 (6.3%)

B: different outcomes			
CR with full renal recovery	21 (95.5%)	6 (50%)	7 (43.8%)
Adverse outcome			
CRF	0	4 (33.3%)	5 (31.3%)
Other organ complications	0	1 (8.3%)	2 (12.5%)
Death	1 (4.5%)	1 (8.3%)	2 (12.5%)

C: anti-FH titer in relation to disease outcome in the anti-FH-positive aHUS			
Disease outcome	Complete remission (*n* = 6)	Adverse sequela/deaths (*n* = 6)	*p* value
Anti-FH titer (mean ± SD) in AU/ml	3399.2 ± 3487.9	3481.7 ± 2365.1	0.9

PEX: plasma exchange, FFP: fresh frozen plasma, IS: immunsupressants, CR: complete remission, and CRF: chronic renal failure. Analysis was done using chi-square test.

## Data Availability

The datasets generated during and/or analyzed during the current study are available from the corresponding author on reasonable request.

## References

[1] Brocklebank V., Wood K. M., Kavanagh D. (2018). Thrombotic microangiopathy and the kidney. *Clinical Journal of the American Society of Nephrology*.

[2] Jacobs E., Ortiz C., Licht C. (2019). The role of complement in the pathogenesis of HUS and the TMA spectrum disorders. *Current Pediatrics Reports*.

[3] Sethi S. K, Raina R., McCulloch M., Bunchman T. E. (2019). *Critical Care Pediatric Nephrology and Dialysis: A Practical Handbook*.

[4] Guo W.-Y., Song D., Song D. (2019). Immunological features and functional analysis of anti-CFH autoantibodies in patients with atypical hemolytic uremic syndrome. *Pediatric Nephrology*.

[5] Schaefer F., Ardissino G., Ariceta G. (2018). Clinical and genetic predictors of atypical hemolytic uremic syndrome phenotype and outcome. *Kidney International*.

[6] Bagga A., Khandelwal P., Khandelwal P. (2019). Hemolytic uremic syndrome in a developing country: consensus guidelines. *Pediatric Nephrology*.

[7] Besbas N., Karpman D., Landau D. (2006). A classification of hemolytic uremic syndrome and thrombotic thrombocytopenic purpura and related disorders. *Kidney International*.

[8] Valoti E., Alberti M., Iatropoulos P. (2019). Rare functional variants in complement genes and anti-FH autoantibodies-associated aHUS. *Frontiers in Immunology*.

[9] Sénant M., Dragon-Durey M. (2019). Anti-factor H autoantibodies assay by ELISA. *Autoantibodies*.

[10] Mousavi S. F., Fatemi S., Siadat S. D. (2016). Development and optimization of a homemade ELISA kit for detection of antibodies against Haemophilus influenzae type b. *Jundishapur Journal of Microbiology*.

[11] Ariceta G., Besbas N., Besbas N. (2009). Guideline for the investigation and initial therapy of diarrhea-negative hemolytic uremic syndrome. *Pediatric Nephrology*.

[12] Giner T., Józsi M., Hofer J. (2014). Complement factor H-Antibody-Associated hemolytic uremic syndrome: pathogenesis, clinical presentation, and treatment. *Seminars in Thrombosis and Hemostasis*.

[13] Sinha A., Gulati A., Saini S. (2014). Prompt plasma exchanges and immunosuppressive treatment improves the outcomes of anti-factor H autoantibody-associated hemolytic uremic syndrome in children. *Kidney International*.

[14] Puraswani M., Khandelwal P., Saini H. (2019). Clinical and immunological profile of anti-factor H antibody associated atypical hemolytic uremic syndrome: a nationwide database. *Frontiers in Immunology*.

[15] Brocklebank V., Johnson S., Sheerin T. P. (2017). Factor H autoantibody is associated with atypical hemolytic uremic syndrome in children in the United Kingdom and Ireland. *Kidney International*.

[16] Moore I., Strain L., Pappworth I. (2010). Association of factor H autoantibodies with deletions of CFHR1, CFHR3, CFHR4, and with mutations in CFH, CFI, CD46, and C3 in patients with atypical hemolytic uremic syndrome. *Blood 14*.

[17] Medjeral-Thomas N., Pickering M. (2016). The complement factor H-related proteins. *Immunological Reviews*.

[18] Durey M.-A. D., Sinha A., Togarsimalemath S. K., Bagga A. (2016). Anti-complement-factor H-associated glomerulopathies. *Nature Reviews Nephrology*.

[19] Yoshida Y., Kato H., Ikeda Y., Nangaku M. (2019). Pathogenesis of atypical hemolytic uremic syndrome. *Journal of Atherosclerosis Thrombosis*.

[20] Noris M., Remuzzi G. (2009). Atypical hemolytic-uremic syndrome. *New England Journal of Medicine*.

[21] Holmes L. V., Strain L., Staniforth S. J. (2013). Determining the population frequency of the CFHR3/CFHR1 deletion at 1q32. *PLoS One*.

[22] Fremeaux-Bacchi V., Fakhouri F., Garnier A. (2013). Genetics and outcome of atypical hemolytic uremic syndrome: a nationwide French series comparing children and adults. *Clinical Journal of the American Society of Nephrology*.

[23] Nozal P., López-Trascasa M. (2016). Autoantibodies against alternative complement pathway proteins in renal pathologies. *Nefrologia*.

[24] Togarsimalemath S. K., Si-Mohammed A., Puraswani M. (2018). Gastrointestinal pathogens in anti-FH antibody positive and negative Hemolytic Uremic Syndrome. *Pediatric Research*.

[25] Lee J. M., Park Y. S., Lee J. H. (2015). Atypical hemolytic uremic syndrome: Korean pediatric series. *Pediatrics International*.

[26] Scully M., Hunt B. J., Benjamin S. (2012). Guidelines on the diagnosis and management of thrombotic thrombocytopenic purpura and other thrombotic microangiopathies. *British Journal of Haematology*.

[27] Khandelwal P., Gupta A., Sinha A. (2015). Effect of plasma exchange and immunosuppressive medications on antibody titers and outcome in anti-complement factor H antibody-associated hemolytic uremic syndrome. *Pediatric Nephrology*.

[28] Song D., Liu X., Liu X.-r. (2017). The clinical and laboratory features of Chinese Han anti-factor H autoantibody-associated hemolytic uremic syndrome. *Pediatric Nephrology*.

[29] Gurjar B. S., Manikanta Sriharsha T., Bhasym A. (2018). Characterization of genetic predisposition and autoantibody profile in atypical haemolytic-uraemic syndrome. *Immunology*.

